# Accelerating Haplotype-Based Genome-Wide Association Study Using Perfect Phylogeny and Phase-Known Reference Data

**DOI:** 10.1371/journal.pone.0022097

**Published:** 2011-07-15

**Authors:** Yungang He, Cong Li, Christopher I. Amos, Momiao Xiong, Hua Ling, Li Jin

**Affiliations:** 1 Department of Computational Genomics, CAS-MPG Partner Institute for Computational Biology, Shanghai Institutes for Biological Sciences, Chinese Academy of Sciences, Shanghai, China; 2 Key Laboratory of Computational Biology, CAS-MPG Partner Institute for Computational Biology, Chinese Academy of Sciences, Shanghai, China; 3 Department of Epidemiology, University of Texas MD Anderson Cancer Center, Houston, Texas, United States of America; 4 Human Genetics Center, University of Texas School of Public Health, Houston, Texas, United States of America; 5 State Key Laboratory of Genetic Engineering and Ministry of Education Key Laboratory of Contemporary Anthropology, School of Life Sciences and Institutes of Biomedical Sciences, Fudan University, Shanghai, China; 6 Center for Inherited Disease Research, Johns Hopkins University, Baltimore, Maryland, United States of America; Aarhus University, Denmark

## Abstract

The genome-wide association study (GWAS) has become a routine approach for mapping disease risk loci with the advent of large-scale genotyping technologies. Multi-allelic haplotype markers can provide superior power compared with single-SNP markers in mapping disease loci. However, the application of haplotype-based analysis to GWAS is usually bottlenecked by prohibitive time cost for haplotype inference, also known as phasing. In this study, we developed an efficient approach to haplotype-based analysis in GWAS. By using a reference panel, our method accelerated the phasing process and reduced the potential bias generated by unrealistic assumptions in phasing process. The haplotype-based approach delivers great power and no type I error inflation for association studies. With only a medium-size reference panel, phasing error in our method is comparable to the genotyping error afforded by commercial genotyping solutions.

## Introduction

The availability of inexpensive platforms for performing dense single nucleotide polymorphism (SNP) analysis makes it possible and affordable to conduct GWAS of complex diseases. Nearly 800 risk SNPs have been reported from over 600 genome-wide association studies in the past years [1].

Power to detect disease susceptibility loci is an essential consideration in the design of GWAS. Researchers have compared the power of single-SNP and haplotype-based association analysis in different genetic scenarios. The benefit of including haplotype-tagging SNPs, especially those based on a cluster of 2–3 SNP markers, has been well recognized after the discovery of “block-like” linkage disequilibrium (LD) pattern in human genome [2]. Theoretical studies demonstrated that the use of multi-allelic haplotypes significantly improved the power and robustness of association studies [3]. This theoretical analysis has been well supported by association studies for many different traits. Haplotypes conferring high susceptibility were identified for schizophrenia, nicotine dependence and macular degeneration for example [4]–[6].

However, two technical issues may hinder the implementation of multi-allelic haplotype-based analysis in GWAS. On the one hand, the inference of haplotypes, also known as phasing, is time-consuming given the huge number of genetic markers in GWAS. Numerous efforts have gone into developing time-saving algorithms, such as fastPHASE, Haplotyper, Hap, Beagle, MACH and 2SNP etc., for example [7]–[12]. Most of these programs are still difficult to apply for routine use in GWAS, although Beagle has shown preliminary success [13]. PLINK implemented a standard expectation maximization algorithm to conduct haplotype-based analysis but phasing quality of the standard EM algorithm is still unknown when applied to GWAS data [14]. On the other hand, HWE and other assumptions in phasing process may lead to problems in GWAS such as decreased phasing quality and statistical bias although some phasing algorithms showed robustness to departures from the assumptions [12], [15], [16]. Due to the nature of the sampling strategy in case-control studies, the problems that result from such assumptions may emerge with the markers surrounding high-risk loci. Permutation supplies a possible solution to eliminate bias in statistical tests but it entails an even more prohibitive time cost. The development of a fast algorithm with robustness to the departure from assumptions would greatly benefit statistical test and data mining in GWAS.

In this report, we present an efficient method utilizing pre-selected SNP clusters and reference phylogeny to improve data analysis in GWAS. This efficient approach delivers a great power than single-SNP analysis and introduces little bias to the statistical analysis.

## Results

### Accuracy of haplotype reconstruction from phase-known reference panel

We proposed a sampling model to study sampling process of a phase-known reference panel. The reference panel included haplotype information from dozens or hundreds of individuals. As the haplotypes observed in the reference panel is a subset of all the existing haplotypes of a natural population due to limited samples in the reference panel, we classify all the existing haplotypes into two groups, named “observed” and “unobserved” groups. The haplotypes presented in the reference panel are “observed” haplotypes and the absent haplotypes are “unobserved” haplotypes.

Given p as overall frequency of the “unobserved” haplotypes in a natural population, a two-step sampling process can generate a subpopulation (size n) with or without the “unobserved” haplotypes. In the first step, n random number {ζ_1_, ζ_2_, …ζ_i_, …ζ_n_} are generated with uniform distribution in the range from 0 to 1. In the second step, chromosomes are sampled sequentially from the population following the rules below.

- When **ζ**_i_>p, a chromosome carrying “observed” haplotype was added to the sample set.
- If **ζ**_i_≤p, a chromosome carrying “unobserved” haplotype was added.

Under the rules, none of the “unobserved” haplotypes appears in the sample set when min{**ζ**_i_}>p.

Overall frequency of “unobserved” haplotypes in natural population is unknown. Given 1-p>>p, we treat E(**ζ**_min_) as an upper bound of p when size of the phase-known reference panel (n) is large.

The cumulative distribution function (CDF) of min{**ζ**_i_} is
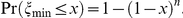



The expectation of min{**ζ**_i_} can be calculated in
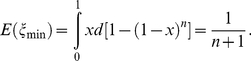
(1)


Using the E(**ζ**_min_), phasing performance of our method could be explored in general scenarios for GWAS. In the investigation, for each SNP cluster in our phasing process (see method section for details), we define that a genotype is “permitted” genotype if it is a combination of two “observed” haplotypes.

We grouped all genotypes in GWAS study into three categories. The “permitted” genotype was considered as “phase-known genotype” because their haplotypes were fully determined by perfect phylogeny of the observed haplotypes. Most of the rest genotypes in GWAS data were a combination of one observed haplotype and one unknown haplotype. They are considered as “predictable genotype” in our study because we have introduced a phasing rule to handle this situation (see method for details). Only a small proportion of the genotypes in GWAS data are the combination of two unknown haplotypes. We considered those genotypes as “phase-unknown genotypes”.

When chromosome number (size n) of the reference panel is large, the *E*(**ζ**_min_) is close to p. Proportions of the three genotype categories in GWAS data thus could be estimated in *E*(ζ_min_)^2^, 2*E*(ζ_min_)(1−*E*(ζ_min_)) and (1−*E*(ζ_min_))^2^ with the assumption of Hardy-Weinberg equilibrium, respectively. In nature of our approach, correct haplotype identifications in our phasing process must be greater than the number of “phase-known genotypes” and slightly less than the sum number of “phase-known genotypes” and “predictable genotypes”.

**Figure 1:**
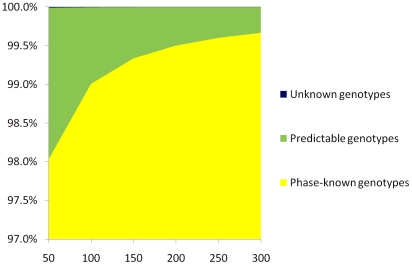
Proportion of “phase-known genotypes” and “predictable genotypes” growths with the increase of reference panel size. The y-axis show proportion of different genotype categories in GWAS data; number of individuals involved in reference panel was shown on x-axis. “unknown genotype” is only a very small proportion.

Proportion of the genotype categories changed with the change of reference population size because upper bound of unobserved haplotype proportion *E*(**ζ**_min_) is determined by the reference panel size in Equation 1 (Figure 1). The result has indicated that the performance of our method continuously improves with an increase of reference panel size. The performance could fully satisfy the needs of haplotype-based association studies with only a middle-size reference panel.

To evaluate our method in real-world data, we checked phasing errors in a 6-fold cross-validation using the phase-known CEU dataset of HapMap Project (see method for details). Proportions of correct phasing ranges from 99.61% to 99.63% in the validations using reference panel with 50 unrelated individuals (Table 1). The proportions are concordant with the above analysis upon the proposed sampling model. This result has confirmed the above conclusion that our method is accurate in haplotype reconstruction with a middle-size reference panel.

**Table 1:** Performance in 6-fold cross-validation (CV) using phased CEU data of HapMap Project.

	cv1	cv2	cv3	cv4	cv5	cv6
Total haplotypes	6115180	6118780	6112460	6115120	6115720	6113780
Wrong haplotypes	23656	23104	23495	22888	23347	22751
Unknown haplotypes	14532	14849	14979	14455	14148	14867
Phasing error (%)	0.39	0.38	0.38	0.37	0.38	0.37
Unknown haplotype (%)	0.24	0.24	0.25	0.24	0.23	0.24
Correct phasing (%)	99.61	99.62	99.62	99.63	99.62	99.63

Proportion of unknown haplotypes (about 0.25%) in the assessment is less than that was expected, *E*(**ζ**_min_)≈0.9% when 50 individuals were involved in reference panel (Equation 1). This could be due to phasing error on the predictable genotypes. In this scenario, one genotype has two (or more) possible explanations for its haplotypes. For example, the genotype could be explained as either combination of two observed haplotypes or one observed haplotype with one unknown haplotype. In our method, we always chose the first solution (two observed haplotypes) even if the later one is actually correct (see method section for details). Most of the phasing errors from our method were because of the incorrect choices.

### Performance in association study with simulation data

Recent progress in algorithm development has greatly improved the performance of haplotype inference. MACH, Beagle and 2SNP etc. declared high efficiency and haplotype inference in “PHASE quality”. We tested all the three representative algorithms on ten simulation data sets (see method for details). Beagle finished each of the data sets in ∼2.5 hour in a single Intel® Xeon 2.5 GHz processor core and 1.5G RAM, whereas MACH and 2SNP finished phasing process for one data set on the same computational platform in 68 and 75 hours, respectively. Due to their large timing cost, MACH and 2SNPs were not considered in following comparison because they are unlikely to be more competitive in GWAS than Beagle. Considering GWAS involves markers ∼25–50 times greater than the simulation data sets, Beagle is the appropriate phasing solution for GWAS among the three candidates.

Compared to the total timing cost in Beagle (25 hours for 10 data sets), Haplominer, the program implemented our algorithm, took only 2.6 hours to finish both the phasing process and association analysis on all 10 data sets. The analysis is almost 10 times faster than that of Beagle.

**Figure 2:**
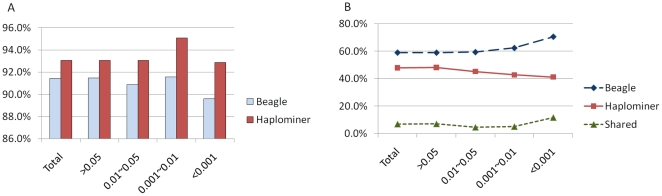
Performance of Haplominer and Beagle-based approach in association study with simulated data. A. Percentage of accordant p-values in the valuation with simulation data. B. Contributions of different approaches to the total discordant p-values.

We compared accordant rate of p-values between different approaches using standard haplotypes and reconstructed haplotypes (see method for details). Given p-value from the standard haplotypes as a reference, higher accordant rate indicates more reliable performance. Our method outperformed Beagle in the evaluation. The accordant rate in Haplominer approach is 2% higher than that in Beagle approach (Figure 2a). More importantly, the accordant rate in the Haplominer approach held constant for markers with different significance levels, while the performance of Beagle decreased with the p-values of markers. Its contribution increases from 59.0% to 70.5% to the total discordant p-values (pooling all discordant results from both the approaches) when significant level of the markers decreases from above 0.05 to below 0.001 (Figure 2b). It was noticed that the overlap of discordant results from different methods is relatively small (from 4.6% to 11.8%, Figure 2b). It is therefore possible to minimize power loss due to the phasing errors by conducting analysis using both approaches.

Bivariate correlation analysis shows that p-values from both Haplominer and Beagle approaches are well correlated with p-values from the standard haplotype sets. Pearson's coefficient is 0.999 between Beagle approach and the approach with standard haplotypes. It is slightly higher than the coefficient (0.998) between Haplominer approach and the standard haplotype approach. Beagle tends to make errors on fewer individuals than Haplominer does though Haplominer makes errors on fewer SNP clusters than Beagle.

**Figure 3:**
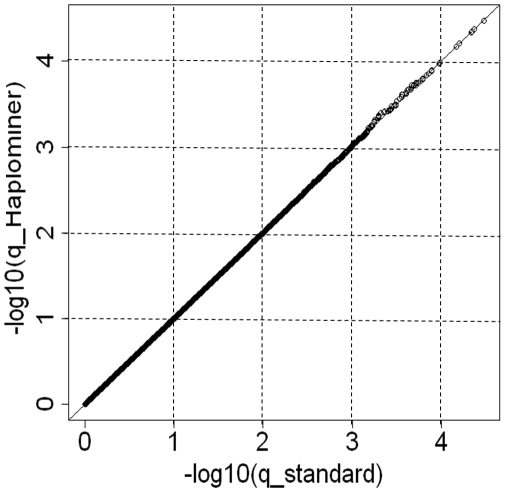
Q-Q plot of p-values in −log10 scale. Quartile of p-values from our approach was shown on y-axis and x-axis presented quantile of p-values from analysis on raw haplotype data.

The other major concern is potential bias in statistical test of GWAS, which can be conveniently examined using QQ-plot. We plotted quantiles of p values from our method against the quantiles of p values from standard haplotypes (Figure 3). The quantiles fit each other well. No obvious statistical bias was observed in the results from our method.

### Application to real GWAS data

Power of multi-allelic haplotype in association study has been well investigated in both theoretical analysis and computer simulation [3], [17]. In this report, we evaluated overall performance of our method on both phasing and statistical test in a GWAS data set from a rheumatoid arthritis (RA) study [18]. After initial screen, 485,841 SNPs and 354,010 SNPs clusters were used as single-SNP and haplotype markers in statistical test for association, respectively. To account for multiple testing, P<1.0×10^−7^ was used as a universal threshold for declaring significance in both single-SNP and haplotype-based analysis.

**Figure 4:**
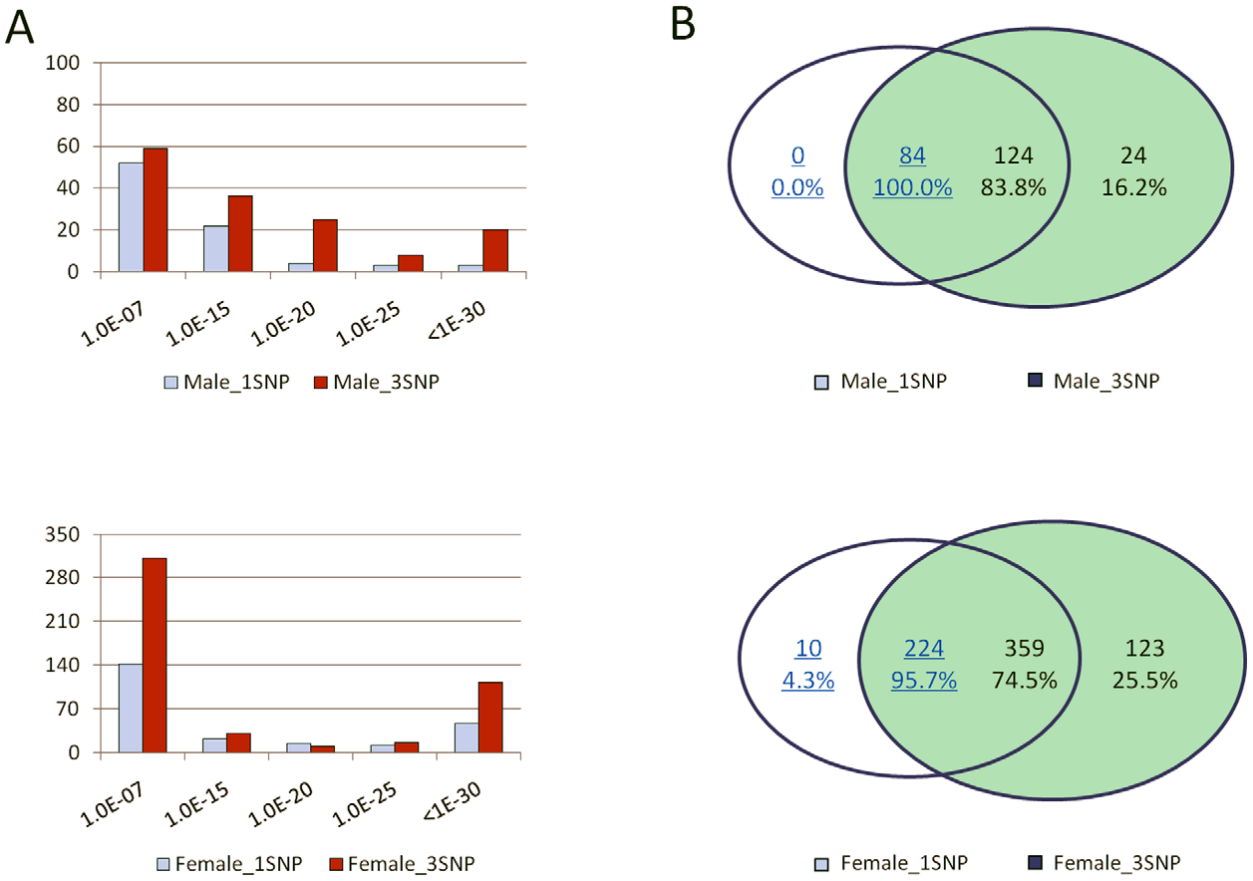
Comparison of results from single-SNP and Haplotype-based analysis. A. Histograms of single SNPs and SNP clusters with p-value less than 1.0×10^−7^ in analyses for male and female data sets, respectively. B. Venn Diagrams showed the sharing of significant single SNPs and SNP clusters in association studies for male and female data sets, respectively. A single-SNP or haplotype finding was shared with the other approach when the significant SNP appeared in any of the significant SNP clusters or any SNP of the cluster appeared in findings of single-SNP analysis, respectively.

In the female only GWAS (with 633 cases and 846 controls), 234 single-SNP markers and 482 SNP clusters showed significant association with RA in single-marker analysis and haplotype-based analysis, respectively. In the male only GWAS (with 226 cases and 339 controls), the numbers of associated single-SNP markers and SNP clusters are 84 and 148, respectively. It is obvious that haplotype-based association analysis in our approach revealed more significant associations with rheumatoid arthritis than single SNP-based association analysis in both studies (Figure 4A). A large proportion of the significant loci in haplotype-based association studies (25.5% for females and 16.2% for male; Figure 4B) were missed by single-SNP analyses (no SNPs from the clusters were significant in the single-SNP analysis). In contrast, only 4.3% and 0.0% of the single SNP findings were missed in the haplotype-based analyses (Figure 4B).

**Figure 5:**
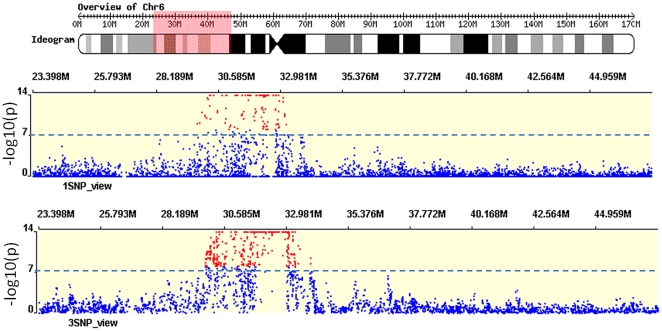
Haplotype-based analysis is more powerful than single-SNP analysis. Upper panel shows results from single-SNP analysis; lower panel presents results of haplotype-based analysis.

171 single SNPs and 628 SNP clusters on Chromosome 6 yielded positive associations with RA in analysis for full RA data set (without considering gender). Given the fixed rejection threshold, disease prevalence, effect size of disease allele, LD and allele frequencies etc., our approach detected more markers in association with disease status around known disease loci (Chromosome 6p near the HLA region) in statistical tests, indicating that haplotype-based association studies have greater power than single SNP-based association studies (Figure 5). Our haplotype-based analysis outperformed the single-SNP-based analysis in the real GWAS data set.

In efficiency, Haplominer, the C++ program implementing our method, finished the above analysis in 5.5 hours (on a single Intel® Xeon 2.5 GHz processor core and 2G RAM, Windows XP 64bit OS). It took Beagle 84 hours to finish only the phasing jobs for the same data sets on the same platform.

To evaluate false positive discovery in our findings, we used WTCCC RA data with imputed genotypes as an independent replication for the genetic associations on Chromosome 6. Results showed that 90.4% of the declared associations (568 in 628 SNP clusters) were successfully replicated in WTCCC data set by haplotype-based analysis while only 83.0% of the declared associations (142 in 171 SNPs) were replicated by single-SNP analysis. This finding indicated that haplotype-based study is more robust than single-marker analysis. This is a favorable feature since poor replication can be a serious problem in GWAS.

## Discussion

Models describing genotyping errors have been well established, but models of phasing error remain relatively scarce. For case-control studies of genetic association, researchers have found that, for a particular error model, known as model GLHO (Gordon, Liu, Heath and Ott presented), there is no increase in type I error due to errors in genotyping [19]. Study of the GLHO model benefits our understanding to phasing error. In our method, phasing errors are introduced to different haplotypes of different SNP clusters in the similar manner of genotyping errors in the model GLHO under the assumptions of random mating and sampling. Phasing errors in our method, therefore, do not increase type I error in GWAS when haplotypes are treated as alleles of multi-allele markers. The conclusion is concordant with our observation in QQ plot (Figure 3).

Both genotyping error and phasing error impact GWAS. Comparison of identical SNPs genotyped by different platforms provides an approximation of genotyping errors in GWAS. It has been reported that accordant rate of genotypes between Illumina and Affymetrix arrays is as high as 99.22–99.73% [20]. Rate of correct phasing is 99.61–99.63% in our method with only a middle-size reference panel (table 1). The rate will continuously improve with increasing size of phase-known reference panel in our approach. The phasing accuracy of our method is the same good as genotyping accuracy of current genotyping platforms. The phasing error is not a technical lesion in the haplotype-based GWAS.

Some sophistic methods have been developed to use haplotypes in association study, such as Blossoc, CLADHC, Margarita and AncesHC etc. [21]–[24]. However, most of the complicate methods would demand a noticeable CPU times for computation when the methods worked on a huge amount of data. The methods are therefore less efficient than our method in GWAS. Haplominer, the same as Beagle and PLINK, directly used SNP clusters as multi-allelic pseudo markers in association studies. Statistical tests (Fisher's exact test or Pearson's chi-square test etc.) in those methods are easy to compute and powers of the classic statistical tests are well known in statistical theory and disease models.

Algorithms for haplotype reconstruction were developed for a variety of purposes in the past two decades though tasks of the algorithms look like similar. In 1990s, both numbers of individuals and genetic markers are generally limited in works of haplotype reconstruction. Efforts for algorithm development focused on improving accuracy in phasing task with limited data size. In 2002, the partition and ligation strategy was introduced to phasing algorithms to handle large amount of genetic markers [12]. Soon after that, with progresses in GWAS, efficiency as well as accuracy became a focus for algorithm development. MACH is one of the algorithms with high efficiency and accuracy when working on large data set [11]. However, only Beagle was developed specifically to handle GWAS data and had higher efficiency than other algorithms in many scenarios [10]. Most of the aforementioned methods paid more attention on haplotype inference than that on association study. In this report, we introduced an efficient method for haplotype-based GWAS. Our purpose is to supply the most efficient solution for haplotype-based association study with thousands of individuals and millions of markers rather than providing a method for haplotype reconstruction. Therefore, our approach and the aforementioned phasing algorithms are running on different tracks with different purposes. In particular, the algorithm we developed will work best in areas of high linkage disequilibrium.

A phase-known reference panel was utilized in our method. Published phasing algorithm, such as PHASE, produced reliable haplotypes for family data with error rate 0.16% or smaller [25]. Haplotype information from trios of HapMap project would serve well as haplotype references in our method. However, phasing errors increased when the existing algorithms worked on genotypes of independent individuals [25]. The increase of phasing errors may be critical when statistically phased haplotypes of independent individuals are used as haplotype references. We therefore examined the possibility of using statistically phased haplotypes of independent individuals as a phase-known reference panel. Utilizing the simulation data and evaluation methods that were used to evaluate performance of our method above (results presented in Figure 2 & 3), our analysis showed statistically phased haplotypes (from Beagle) had performance very similar with the standard haplotypes. 98.54% of total results (223634 of 226958 p-values) are identical to the above results in association studies yield by using standard haplotypes as references. Statistically phased haplotypes could serve well as haplotype references in our approach.

In summary, we supplied an efficient approach to haplotype-based GWAS. The approach delivers great power and no type I error inflation to association studies. To the best our knowledge, it is one of the most efficient approaches that have been published.

## Materials and Methods

### Approach to haplotype-based GWAS

The objective of the proposed method is to replace single SNP marker with haplotypes of multiple SNPs for GWAS analysis. This method involves three steps: SNP selection, phasing and statistical test. The method was implemented in a C++ program, *Haplominer*. Source codes of the program and related files could be downloaded from website of sourceforge.net (http://haplominer.sourceforge.net) or authors' website (http://www.picb.ac.cn/~yunganghe/haplominer). Details of the method were addressed blow.

#### Select SNP clusters using a reference population

We first identify SNPs with criteria of perfect phylogeny whose haplotypes can be determined for association studies without invoking recombination [26]. These SNPs are close to each other but not necessarily contiguous. To boost speed of the identification and avoid the complications involved in haplotype inference, SNP selection is therefore suggested to be done using a reference population with available haplotype information. For a proper application of the method, it is important to ensure the ethnicity of the population for SNP selection should match that of case-control samples in an association study. In this report, we used CEU data set from the International HapMap Project for SNP selection as an example, in which highly reliable haplotypes were inferred from trio samples.

For each given SNP, additional SNPs were selected based on haplotypes of the reference population, to form a SNP cluster whose haplotypes could be inferred without invoking recombination [26]. The SNP selection requires a given size of genomic region (typically 5–30 kb) and a predetermined maximum number of SNPs (typically 2–4 SNPs). An excessive number of SNPs may result in a reduction of statistical power [3]. The additional SNPs can be achieved by searching exhaustively all allowed SNP clusters in the working region with the reference population, and the cluster, therefore SNPs, yielding maximum entropy was selected. It is of course important to select only the SNPs that are shared between the reference population and the samples (including both cases and controls) for GWAS analysis.

This exercise would yield a cluster of selected SNPs (or a SNP combination) for each given SNP. Each SNP, which could now be replaced by the haplotypes of the corresponding SNP cluster, would be interrogated individually in the GWAS analysis. In this report, the haplotypes observed in the reference population are also referred to as “observed” haplotypes, otherwise “unobserved” haplotypes.

#### Haplotype identification in GWAS samples

For a SNP cluster associated a given SNP, genotypes of a GWAS sample have only one permitted solution (consisting of two observed haplotypes) in the inference of haplotypes, theoretically. In other words, all GWAS genotypes, including cases and controls, can be deconvoluted uniquely into the observed haplotypes if the reference population carries all the haplotypes in the GWAS samples, typically when the reference population is large enough. However, when the reference population is not sufficiently large, in reality, we may encounter unpermitted genotypes (consisting at least one unobserved haplotypes) in GWAS samples, though rarely [27].

**Figure 6:**
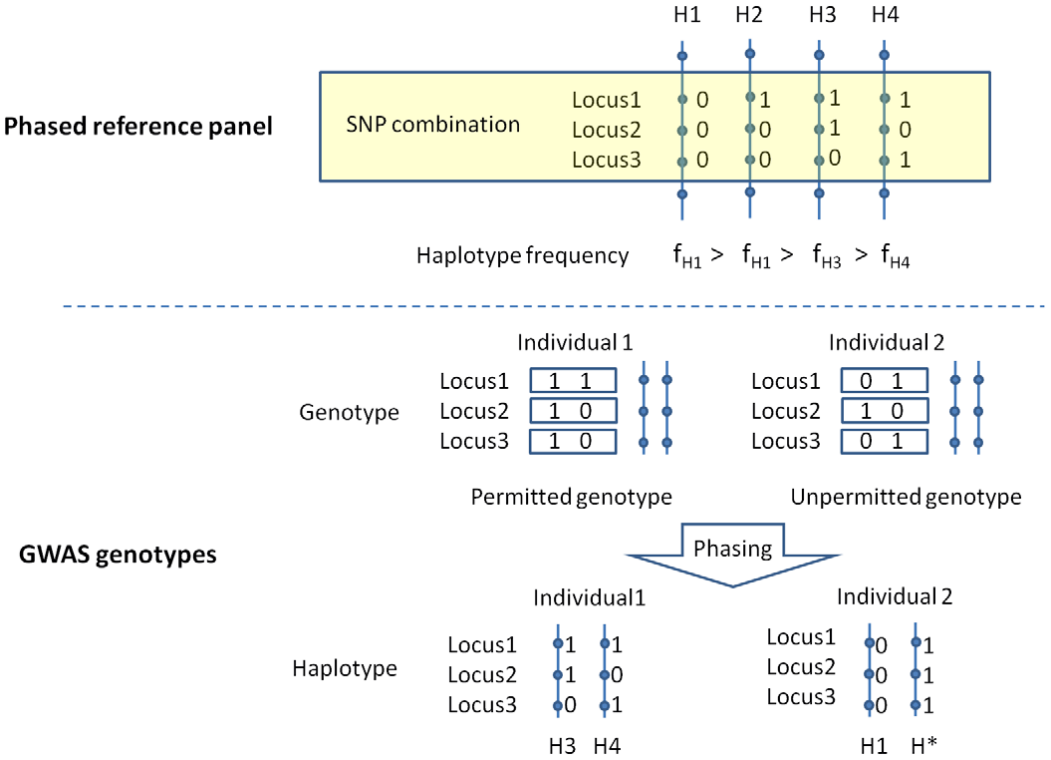
An example for haplotype identification with selected SNP cluster and phase-known reference population.

Most of the GWAS samples can be deconvoluted into observed haplotypes (Figure 6). When this cannot be achieved, two scenarios may arise. (1) The unpermitted genotypes can be dissected into one observed haplotype and its' complementary haplotype. However, when multiple solutions are possible, the observed haplotype with higher frequency in the reference population is chosen. (2) The unpermitted genotypes can only be explained by two unknown haplotypes. The unknown haplotypes in both the scenarios do not damage further analysis because all the unknown haplotypes will be pooled together for Pearson's chi-square test upon contingency table.

The phasing procedure could be presented below in pseudo code.

#DEFINE an observed haplotype is a haplotype that was observed in the reference panel for a given SNP cluster
#DEFINE a permitted genotype is a genotype that could be explained as a combination of two observed haplotypes
#DEFINE unknown haplotypes are a group of haplotypes which could not be identified in our phasing process. They were pooled together in statistical test as one of the haplotype category.

IF the genotype of an individual for a given SNP cluster is a permitted genotype

 Unravel the genotype into two observed haplotypes

ELSE

 IF the genotype could be explained as a combination of one observed haplotype and its' complementary haplotype

   Unravel the genotype to one observed haplotype and its' complementary haplotype

 ELSE

  Explain the genotype as the combination of two unknown haplotypes

 END

END

#### Statistical test

Pearson's chi-square test is performed on a contingency table with haplotype data in association study. In the test, for each cluster with *n* SNP markers, counts of haplotypes in case and control groups were organized into a *m* by 2 contingency table (for example, m = n+2 or m = n+1 for scenarios with or without unobserved haplotype). For each haplotype marker, all unobserved haplotypes were pooled together to reduce degree of freedom in the Pearson's chi-square test.

### Phasing performance

#### Evaluation with HapMap data

In order to evaluate phasing performance of our method in real genotyping data, we conducted cross-validations in the phase-known CEU data set from HapMap Phase II. 60 unrelated individuals of the CEU panel were randomly permutated and then assigned into 6 groups with 10 individuals each. In each validation, haplotypes of 10 individuals in one of the groups were used as standard to evaluate phasing quality while haplotypes of other 50 individuals served as the reference panel. The validation began by selecting a set of SNP clusters from the reference panel in the same manner described above with a maximum cluster size of 3 and a window size of 20 kb. In our method, we reconstructed haplotypes for genotypes of SNP clusters of the 10 individuals then compared to the standard haplotypes.

### Performance of our method on GWAS data

#### Performance on simulation data

In the evaluation, ten data sets were generated in MaCS (http://www-hsc.usc.edu/~garykche/) under the frame of coalescent theory [28]. We assumed Ne = 5,000, μ = 2×10^−8^ per bp, and r = 1.2×10^−8^ per bp. This translates to a scaled mutation rate and recombination rate (scaled in units of 4Ne generations) of 10,000 and 6000 for a 25-Mb region.

Each data set contains 2,200 chromosomes, 200 of which were used as a phase-known reference panel. The rest 2,000 chromosomes were randomly assigned into case or control group with 1,000 chromosomes each. In each group, genotypes of one individual were determined by joining two randomly chosen chromosomes. Only SNPs with minor allele frequency (MAF) larger than or equal to 0.05 were used in further evaluation.

Our method and three representative phasing algorithms (MACH: http://www.sph.umich.edu/csg/abecasis/MACH/, Beagle: http://www.stat.auckland.ac.nz/~bbrowning/beagle/beagle.html and 2SNP: http://alla.cs.gsu.edu/~software/2SNP/) were evaluated in the simulation data set. For a high phasing quality, the simulated genome fragments were phased as a whole (without cutting to pieces) in the three representative phasing algorithms. MACH and 2SNP did not show competitive efficiency in the initial evaluation for timing cost. Only Beagle and our method were included in further comparisons.

Using the simulation data, we selected SNP clusters and calculated p-values for each of the SNP clusters in our method. Each of the clusters includes 3 SNPs from a working window of 5,000 bp in size. Raw haplotypes or Beagle-generated haplotypes for the selected SNP clusters were used to organize the simulated case-control data into cross tables. Pearson's chi-square test was performed on the cross tables from a different approach to corresponding p-values.

To rate performances in association study directly, we compared accordant rate of p-values between our method and Beagle-based approach. In this report, the accordant rate of a specific approach is a proportion of its statistical tests that gave the same p-value as the corresponding tests using raw haplotypes.

#### Performance on real data

A GWAS data set for rheumatoid arthritis (RA) from the North American Rheumatoid Arthritis Consortium (NARAC) was used to evaluate our method [18]. SNP clusters were selected in the phase-known CEU data set from HapMap Phase II with a given maximum cluster size of 3 and a window size of 20 kb. Before any further analysis, we conducted a multi-level data clean in PLINK [14]. Individuals having cryptic family relationships or a rate of genotype missing larger than 5% were excluded from association analysis. SNP markers having minor allele frequency less than 1% or missing data more than 5% were excluded. Furthermore, all involving loci passed a statistical test for HWE with p-value larger than or equal to 1×10^−5^. After the data cleaning, 502,763 SNPs remained from 859 cases and 1185 controls. The data set included 565 males and 1479 females. Both standard single-marker analysis and our haplotype-based analysis were performed on the data set. Positive findings on Chromosome 6 were visualized in WGAViewer to present the power difference between the two approaches [29].

We also validate the positive findings on Chromosome 6 using WTCCC RA data sets [24]. About 80% of SNP markers on Chromosome 6 from above RA genotyping data cannot be found in WTCCC RA data due to the using of different genotyping platforms. Genotype imputation was carried out in Beagle to fill the missing genotypes in WTCCC RA data with HapMap CEU data as a reference. We conducted association analysis on the WTCCC RA data with both real and imputed genotypes using the same approach that has been used for NARAC data analysis. The positive findings from the above NARAC data analysis were checked carefully using the WTCCC RA data for rate of replication.

The institutional review board reviewed and approved the study in accordance with the code of ethics of the World Medical Association (Declaration of Helsinki).
